# A cross-sectional mixed-methods study of nurses’ quality of working life in telenursing and care satisfaction comparing those participating in continuing professional development or not during the previous year

**DOI:** 10.1186/s12912-026-05205-7

**Published:** 2026-08-11

**Authors:** Annica Björkman, Karin Myrberg, Anna Carin Wahlberg, Maria Engström

**Affiliations:** 1 Department of Health and Caring Sciences, University of Gävle, Gävle, Sweden; 2 Department of Neurobiology, Caring Sciences and Society, Karolinska Institutet, Stockholm, Sweden; 3 Department of Nursing Science, Lishui University, Medicine College, Lishui, China; 4 Centre for Research and Development, Uppsala University/Region GävleborgCenre for Research and Development, Uppsala and Gävle, Sweden

**Keywords:** Continuous professional development, Nurses, Quality of working life, Satisfaction with care, Telenursing, Thriving, Workforce sustainability

## Abstract

**Background:**

Rapid changes in healthcare and digitalization underscore the importance of continuing professional development (CPD) in nursing. However, participation is often limited by high workloads, staffing shortages, and insufficient organizational support. Telenursing is a growing field with specific competence requirements, yet previous research indicates demanding working conditions and limited development opportunities. Further evidence is needed regarding CPD participation, its association with work-related outcomes, and nurses’ perceived CPD needs.

**Aim:**

The aim of the study was to compare nurse-rated quality of working life and satisfaction with given care among nurses working in telenursing services who had participated in CPD during the previous year and those who had not. A further aim was to explore nurses’ preferences and perceived needs related to CPD activities.

**Methods:**

A cross-sectional survey among nurses in Sweden’s national healthcare advisory service (1177) measured quality of working life four instruments were used. Emotional thriving was measured using a brief four-item scale. Telenursing working conditions were assessed using a telenursing-specific instrument. Growth opportunities were measured with a six-item factor from the instrument Safety, Communication, Operational Reliability and Engagement and job satisfaction using the four-item Brief Index of Affective Job Satisfaction scale. Satisfaction with given care was measured using the eight-item Satisfaction with Given Care scale. Participation in continuing professional development (CPD) was assessed by self-reported number of CPD days during the past year, including none, and an open-ended question capturing desired areas of competence development.

**Results:**

Nurses who had participated in CPD reported significantly higher emotional thriving, as well as higher ratings for management and collegial support and growth opportunities, compared with those not participating in CPD. No group differences were found regarding satisfaction with given care. Identified CPD needs included strengthened clinical knowledge, reflective and self-directed learning, and improved communication skills.

**Conclusion:**

Participating in CPD during the previous year is associated with more positive work-related outcomes among telenursing nurses such as thriving, growth opportunities, better management and collegial support but not with nurse-sensitive outcomes such as nurse satisfaction with given care. Nurses described clear preferences and needs for clinically relevant, reflective, and self-directed professional development.

**Clinical trial number:**

Not applicable.

**Reporting method:**

This study adhered to the relevant EQUATOR guidelines for observational studies and was reported in accordance with the STROBE. See attached file STROBE_CPD.

**Patient or public contribution:**

The research focused exclusively on healthcare professionals’ experiences and working conditions; therefore, no patient-facing activities were included. However, the study was informed by prior literature and organizational feedback to ensure that the research questions were relevant to clinical practice and service delivery.

## Introduction

The role of nurses is continually evolving in response to shifting demographic health challenges and ongoing medical advancements [1]. To meet these demands, nurses have a professional responsibility to keep their knowledge up to date and engage in lifelong learning [2, 3]. However, obstacles such as heavy workloads, insufficient staffing, lack of funding, and limited managerial support can impede participation in Continuous Professional Development (CPD) [4, 5]. Telephone advice nursing, here after called telenursing exemplifies how technological advancements are evolving in the nursing profession. Since telenursing as a novel care delivery method may require increased CPD, it is notable that nurses often work under demanding conditions with restricted access to professional development [6–8]. Given the critical role of CPD in maintaining competence and enhancing quality of working life, understanding its availability, associated factors, and the specific needs and preferences of nurses is essential. In this study, *quality of working life* is understood as nurses’ self-reported perceptions of their psychosocial work environment and professional situation, including aspects such as emotional well-being, perceived managerial and collegial support, opportunities for professional growth, and contextual working conditions [9, 10].

## Background

CPD in nursing has been defined as: “a life-long process of active participation by nurses in learning activities that assist in developing and maintaining their continuing competence, enhancing their professional practice and supporting achievement of their career goals” [11]. It is a continuous process of enhancing knowledge, skills, and attitudes to improve their ability to provide high-quality patient care [12]. The need for continuous development of knowledge and skills encompasses diverse areas such as clinical practice, administration, and research [2]. Some studies have documented the positive impact of CPD on patient outcomes [13], professional growth [14] and organizational performance [15] and found CPD related to person-centered care [16]. Despite the importance of CPD for nurses, there are several challenges in this area. First, there are methodological limitations in examining the effects on patient outcomes. There is also a growing awareness that the knowledge and competencies acquired through CPD are not necessarily automatically transferred into practice and that this application is influenced by contextual factors [17]. Among the challenges mentioned in a scoping review by Hakvoort and colleagues [4] are the lack of nursing staff development plans, limited career paths, inadequate staffing levels, and insufficient support from colleagues, managers, and employers. Additionally, nurses experience a disconnect between educational activities and their practical application. A meta synthesis [5] showed how the most common perceived barriers to CPD were poor staffing levels, heavy workloads, lack of funding and lack of study time. On the other hand, continuous learning is central in a knowledge intensive sector such as health care, and higher nurse competence have been found related to less stress symptoms among nurses [18] and a more person-centered care [16]. However, methodological challenges remain in demonstrating direct effects on patient outcomes, and there is increasing recognition that the knowledge and competencies gained through CPD are not always readily applied in practice, as their transfer is shaped by contextual factors [17].

Nurses have a professional responsibility to maintain up-to-date competencies and require lifelong learning to effectively operate in an evolving and highly demanding healthcare environment [3]. CPD participation is mandatory for nurses in many countries, such as the UK and the US, whereas in others, including Sweden, it is not required. Motivations for engaging in CPD vary, with younger nurses focusing on career advancement and older nurses prioritizing patient care ([19] Organizational culture shapes the possibility and conditions for nurses CPD as they are depending on organizational support and culture [5, 20]. Nevertheless, continuous learning is crucial in the knowledge-intensive healthcare sector, and higher nurse competence has been linked to reduced stress symptoms and more person-centered care [21].

### Telenursing

In the Swedish context, the term *telenursing* is commonly used to describe nurse-led remote care services. Internationally, similar forms of care are more often referred to as *telehealth*, *virtual care*, or *remote care*. These differences in terminology should be considered when comparing findings across healthcare systems. Telenursing includes all kind of care where nurses, deliver nursing services, triage care seekers need of care and give self-care advice via remote telecommunications such as telephone, video consultations and chat/text consultations [22]. Telenursing is a rapidly expanding field [22]. It is emerging as a critical strategy for optimizing healthcare resources and improving access to appropriate care ([23]. Within the publicly financed primary care system, a national nurse-led telenursing service has become increasingly important in some European countries for managing patient flow and addressing workforce shortages [24]. Telenursing helps manage patient flows by guiding care-seekers before they seek unscheduled care, reducing unnecessary emergency visits and easing the burden on healthcare services. Nurses have reported to having a demanding work where they are exposed to cognitive fatigue and no opportunity for recovery during the work shift [7, 8]. Nurses working in telenursing settings report high levels of various stressors (e.g., higher demands, role conflicts, and work-private life conflicts, lower possibility to influence work, lower feedback and quality of leadership), stress reactions and long-term consequences (e.g., lower job satisfaction and quality of sleep, higher intention to leave and burnout-symptoms) [7, 25–28]. Common stressors also include limited opportunities for professional development, high workloads, time pressure, overtime, and long working hours [29].

Working as a nurse is demanding, knowledge intensive and implies the need for constant knowledge and competence development. Nurses’ ability to develop their competencies is to a large extent influenced by their work environment [30]. As healthcare continuously evolves, with new guidelines, medications, and treatments introduced regularly, nurses must adapt by continually learning new skills [5]. Nurses’ competence has also been found to have a considerable impact on the quality of care and is closely associated with work performance, satisfaction and reduced intention to leave [6, 31]. In this context, *satisfaction with given care* refers to nurses’ own assessments of the quality of care they provide, reflecting their perceived ability to deliver appropriate, safe, and satisfactory care within the conditions of their work. Nurses’ evaluations of the care they give have been widely used as an indicator of perceived care quality and have been shown to be associated with working conditions, professional competence, and organizational [32, 33] support.

In a recent study, we found that good access to structural empowerment, such as opportunities for development and growth, increases nurses’ thriving, which in turn improves work–personal life balance and job performance, while reducing stress symptoms and turnover intentions. Thriving (a sense of learning and vitality) acted as a mediator in the relationships between structural empowerment and these positive outcomes [34]. However, there is limited research on how participation in CPD influences nurses’ quality of working life and satisfaction with care, and on their preferences and needs regarding CPD activities. Addressing this knowledge gap is essential for informing strategies that support nurses’ professional development and help optimize both staff wellbeing and patient care.

### Aim

The aim of the study was to compare nurse-rated quality of working life and satisfaction with given care among nurses working in telenursing services who had participated in CPD during the previous year and those who had not. A further aim was to explore nurses’ preferences and perceived needs related to CPD activities.

## Methods

### Design

The study used a descriptive comparative cross-sectional design with an embedded mixed-method approach [35].

### Study setting and sampling

This study was conducted in Sweden, where telenursing service is organized in regional call-centers [7] staffed by registered nurses. The work environment for these nurses resembles that of a typical call center. Aline with the rest of the Swedish nursing workforce, there is no mandatory requirement for nurses to complete a specific number of hours of CPD. The inclusion criteria were all nurses employed at *SHD 1177* – the Swedish national nurse-led telephone healthcare advice service (*n* = 465). Respective nurse managers provided the email addresses of all employed nurses to the responsible researcher (A.B.). A coded survey invitation was then distributed to all eligible nurses via the SunetSurvey platform in November 2023. Exclusion criteria were nurses on long-term sick leave or maternity leave.

### Measures

The web survey consisted of validated instruments with the opportunity for free-text responses. Emotional thriving was measured using a 4-item scale developed by Adair et al., [36], with five-grade response alternatives. Telenursing working conditions (TWC) were measured using a scale developed specifically for telenursing service [7], with construct validity found acceptable using explorative factor analyses (unpublished data). Growth opportunities were measured with a six-item factor from the Safety, Communication, Operational Reliability and Engagement (SCORE) scale [37], addressing areas such as personal growth, independent thoughts and actions, and feelings of achievement; responses were given on a five-point scale. Job satisfaction was measured using the four-item Brief Index of Affective Job Satisfaction scale [38] with response options ranging from 1 (strongly disagree) to 5 (strongly agree). Satisfaction with given care was measured using the eight-item Satisfaction with Given Care scale [39], with seven-point Likert response options. All instruments have shown good psychometric properties. Participation in CPD was assessed using the question: *“How many days of competence development have you had during the past year?”* with response options to report the number of days, including the option “no days”. In addition, participants were asked an open-ended question: *“What competence development do you wish for?”*, allowing free-text responses where participants could describe desired areas or types of CPD. See Table 1 for description of instruments used in the study.

**Table 1:** Overview of measures and instruments used in the study

Construct	Instrument / items	Response scale	Scoring and interpretation	Reliability in the present study
Emotional thriving	4-item Emotional Thriving scale (Adair et al. [33])	5-point Likert scale (1 = Strongly disagree to 5 = Strongly agree)	Higher scores indicate higher emotional thriving	Cronbach’s α = 0.81
Growth opportunities	6-item factor from the Safety, Communication, Operational Reliability and Engagement (SCORE) scale [34], covering personal growth, autonomy, and sense of achievement	5-point Likert scale (1 = Strongly disagree to 5 = Strongly agree)	Scores transformed to a 0–100 scale; higher scores indicate better growth opportunities	Cronbach’s α = 0.87
Job satisfaction	4-item Brief Index of Affective Job Satisfaction (BIAJS) scale [35]	4-point Likert scale (1 = Strongly disagree to 4 = Strongly agree)	Mean score calculated; higher scores indicate higher job satisfaction	Cronbach’s α = 0.84
Satisfaction with given carte	8-item Satisfaction with Given Care scale [36]	7-point Likert scale (1 = Disagree strongly to 7 = Agree strongly)	Higher scores indicate higher satisfaction with given care	Cronbach’s α = 0.90
Working conditions	14-item Telenursing Working Condition (TWC) scale.	5-point Likert scale (1 = Not at all to 5) = To a very high extent	Higher scores represent better conditions for the factors Preconditions, and Management & colleagues whereas for the factor Obstacles higher scores represent worse conditions	Cronbach’s Alpha and McDonald’s Omega in the present study Preconditions α 0.72; ω 0.72 (6 items), Management & colleagues α 0.68; ω 0.70 (3 items), and Obstacles α 0.81; ω 0.81(5 items).

### Data analysis

Quantitative variables were analyzed using descriptive and comparative statistics (Chi-square test and Mann-Whitney U Test). Visual inspection of histograms for the outcome variables showed non-normal distribution. Effect size was computed for Mann-Whitney U Test [40] According to Funder and Ozer, an r of 0.10 indicates a small effect and 0.20 a medium effect [41].

Free-text replay were analyzed using directed content analysis, a method in which coding starts from predetermined categories based on existing theory or prior research, allowing new categories to emerge as needed [42]. Two authors (A.B. and K.M.) independently familiarized themselves with the data and generated initial codes. These codes were then organized into potential themes, which were reviewed and refined through iterative discussions. After consensus was reached, five distinct themes were identified. The process was reflexive, with an emphasis on transparency and rigor, and all coding and theme development were conducted manually to ensure close engagement with the data. Following the directed analysis, a summative content analysis was applied [42]. This involved counting and summarizing mentions within each category to provide an overview of the most frequently expressed needs. This combined approach offered both qualitative insights and quantitative descriptions of the data. In addition to the directed content analysis, a summative content analysis was applied to descriptively quantify the frequency of mentions within each theme. These counts were used to illustrate the relative emphasis of identified needs, while the primary analytical focus remained qualitative [42].

## Results

### Sample

Of those invited, 187 nurses completed the survey, yielding a response rate of 40.2%. Among the respondents 180 (96.3%) provided a free-text response on the question, “What kind of CPD do you desire?”, which included 122 mentions or suggestions related to their desired CPD in the surveys. For nursing studies, an effect size of small to medium has been suggested. With an effect size 0.40 to 0.50 and a power of 0.80, the required sample size would be 99 to 64 people in each group [43]. In the present study, of the 180 respondents, 124 nurses reported having participated in CPD and 56 did not participate in CPD. A majority of the participants were women (*n* = 169), mean age was 53 years, and they had, in average, worked 25 years as a registered nurse. In general, they had received a median of two CDP days during the past year. There were no statistically significant differences in demographic data between the two groups (CPD yes vs. no). For a description of participants, see Table 2.

**Table 2:** Participant demographic

	Total samplen = 180	Yes CPD, *n* = 124	No CPD, *n* = 56	*P*-value
Competence development, days, mean (SD)	3.8 (3.8) Md 2.0, Q1-3 1.6;4.0	3.8 (3.8) Md 2.0, Q1-3 1.6;4.0	0	
Sex, female/male, n (%)	169/10 (93.9/5.6)	116/7 (93.5/5.6)	53/3 (94.6/5.4)	1.000^a^
Age, years mean (SD), min-max	53.1 (11.1) 27–72	52.2 (10.8) 27–72	55.0 (11.6) 28–71	0.121^b^
Years working as a RN, mean (SD), min-max	24.7 (12.2), 2–49	24.1 (11.8) 3–49	26.2 (13.0) 2–48	0.290 ^c^
Specialist education, yes/no, n (%)	98/81 (54.4/45.0)	66/57 (53.2/46.0)	32/24 (57.1/42.9)	0.747^a^
Working full/part time, n (%)	54/126 (30.0/70.0)	35/89 (28.2/71.8)	19/37 (33.9/66.1)	0.484^a^

When the sum does not add 180 there are internal missing data

Abbreviations: CPD Continuous Professional Development, SD standard deviation, RN registered nurse, ^a^ Chi-Square test, ^b^ independent samples T-test, ^c^Mann-Whitney U Test

### Nurse-rated quality of working life and satisfaction with given care

Comparing the two groups, participated in CPD or not during the year, the CPD yes group reported statistically significantly higher mean scores for thriving compared to non-participants in CPD. Effect Size (ES) reported as r was − 0.20 for the Emotional thriving scale. The CPD yes group also reported statistically significantly higher mean scores for ‘Structural working conditions’ (total score), and in the factor ‘Management and Colleagues’ with ES of *r* = − .16 and *r*=-.20 respectively. For ‘Growth opportunities’ the CPD yes group reported statistically significantly higher mean score than the non-participating group (ES *r* =-.16). These ES can be described as small to medium. For the other variables ‘Job satisfaction’, ‘Nurse satisfaction with given care’ and the factors ‘preconditions’ and ‘obstacles’, the results were non-significant (Table 3).

**Table 3:** Comparison of working life variables and nurse-rated satisfaction with given care (NSC) between the groups participated in competence development during the year yes (*n* = 124) and no (*n* = 56), Mann-Whitney U Test

	CPD Yes	CPD No	*P*-value	Effect size *r*	Total sample *n* = 178–180
	Mean (SD)				Mean (SD)
Emotional Thriving	3.9 (0.7)	3.6 (0.9)	.**006**	− 0.20	3.8 (0.8)
Job satisfaction	3.6 (0.8)	3.4 (0.9)	0.362	− 0.07	3.5 (0.8)
Telenursing work conditions	5.2 (0.7)	4.9 (0.8)	.**026**	− 0.16	5.2 (0.8)
-Management & Colleagues	5.8 (1.2)	5.3 (1.2)	.**006**	− 0.20	5.6 (1.2)
-Preconditions	6.2 (0.6)	6.0 (0.8)	0.205	− 0.09	6.1 (0.7)
-Obstacles^a^	4.2 (1.3)	4.5 (1.3)	0.099	0.12	4.3 (1.3)
Growth Opportunities	63.6 (18.5)	55.2 (24.7)	.**027**	− 0.16	61.0 (20.9)
NSC	5.9 (0.6)	5.9 (0.7)	0.595	0.04	5.9 (0.7)

Abbreviations: CPD Continuous Professional Development, CI Confidence intervals; SD standard deviation, NSC Nurse Satisfaction with given Care. For all scales or factors higher scores mean better outcomes except for the factor obstacles where ^a^ higher scores indicate more obstacles

### Nurses’ preferences and perceived needs regarding CPD activities

The thematic analysis revealed five different themes, all describing what kind of CPD activities the participants desired. See Table 4 for details.

**Table 4:** Description of themes

Themes	*n*	% of total
Clinical knowledge in telenursing	52	42.6%
Reflective and Self-Directed Learning in telenursing	30	24.6%
Communication Skills in telenursing	17	13.9%
Technical and Documentation Skills in telenursing	13	10.7%
Cross-Institutional Exposure outside telenursing	10	8.2%
	*n* = 122 mentions	

### Clinical knowledge in telenursing

Participants expressed a strong need for continuous education through regular lectures and updates on clinical guidelines relevant to telenursing. Commonly mentioned areas included pediatrics, psychiatry, ophthalmology, allergies, and seasonal infections—considered essential for accurate assessments and efficient daily work. Opportunities for skills development were also mentioned as important for introducing variation into an otherwise repetitive work environment, supporting both competence and job satisfaction.

I would like teaching in themes (paediatrics, infections) that support and make me more effective in my work and that improve my assessments.

### Reflective and self-directed learning in telenursing

Structured feedback through supervised call reviews was described as a valuable tool for professional development. Respondents also emphasized the importance of having dedicated time for professional tasks beyond direct patient contact. Suggestions included setting aside weekly time slots or designated administrative days for reviewing updated guidelines, completing documentation, and engaging in self-directed learning.

I would like to be able to learn from one’s own cases – we never find out how it went for the patient, and in some cases this would contribute to learning that otherwise can take a long time to acquire.

### Communication Skills in telenursing

The participants expressed a desired training for managing emotionally challenging interactions, such as conversations involving suicidal ideation and handling aggressive or dissatisfied callers—situations frequently encountered in practice. The growing use of digital tools, including chat and video consultations, highlighted the need for comprehensive training in giving care via written communication in the context of nursing. Additionally, participants identified gaps in English language proficiency for healthcare contexts, noting difficulties with medical terminology and effective communication.

I would have wished to practice with colleagues in “case” format, that is, to go through fictitious or real cases. To discuss how we interpret information and which follow-up questions we ask.

### Technical and documentation skills in telenursing

Several participants highlighted the need for training in effective documentation strategies, expressing interest in learning how to document essential information concisely without producing overly lengthy entries. They also emphasized the importance of developing their IT-related competencies. There was notable interest in gaining deeper insight into decision support tools, especially newly introduced advisory systems. Responses stressed the value of understanding the underlying logic and hierarchical structure of these tools.

We need more technical competence – why it is important to go from top to bottom in the decision support. At present, we have a situation where even experienced colleagues still try to fit every assessment call into the decision support system, regardless of whether the symptom pathway fits or not.

### Cross-institutional exposure outside telenursing

There was a strong interest in job shadowing and clinical observation in other healthcare settings. Participants suggested visits to primary care centers, child health clinics, emergency departments, ambulance services, and emergency dispatch centers. These experiences were viewed as valuable for broadening clinical understanding and enhancing professional competence.

Job shadowing / clinical placement at different workplaces such as the emergency number 112/Emergency Dispatch Centre and the Mobile Primary Care Team with physicians and nurses

## Discussion

The aim of the study was to compare nurse-rated quality of working life and satisfaction with given care among nurses working in telenursing services who had participated in CPD during the previous year and those who had not. A further aim was to explore nurses’ preferences and perceived needs related to CPD activities. The results show that nurses who had participated in CPD reported a more favorable quality of working life in terms of emotional thriving, perceived managerial and collegial support, and growth opportunities. In contrast, participation in CPD was not associated with job satisfaction or satisfaction with given care.

Nurses’ CPD preferences primarily focused on clinically oriented development, particularly enhanced clinical knowledge and opportunities for reflective and self-directed learning, followed by communication skills in digital care, technical and documentation skills, and cross-institutional exposure. Together, these findings highlight nurses’ interest in CPD activities that support both clinical competence and reflective practice within the specific context of telenursing.

CPD is seen by nurses as an indication of being valued by the organization [5]. In our study, participation in CPD was associated with several positive outcomes, including increased thriving, higher ratings of ‘management and colleagues’ and growth opportunities. Thriving, as increased in our study, has in an integrative review by Goh et al. [44], been found linked to improved health, well-being, job performance, work attitudes, and professional development. Given the high demands placed on nurses [7, 8], it is critically important that nurses are provided with the necessary conditions to experience and maintain thriving, a sense of learning and vitality, in their professional roles. One way to support thriving is by ensuring CPD, given its numerous positive effects, including enhanced knowledge and skills, increased professional confidence, and expanded opportunities for career advancement. Furthermore, nurses with CPD reported higher growth opportunities such as personal development, feelings of achievement, and independence in actions.

Keeping up-to-date with healthcare developments is also crucial for safe, effective patient care [5]. However, in our study, nurses with CPD during the year did not report higher satisfaction with given care compared to those not participating in CPD. These findings are not entirely unexpected. Although CPD is often assumed to contribute to improved patient outcomes, establishing a direct and unequivocal link remains methodologically challenging. Previous reviews have shown that evidence linking CPD interventions to patient outcomes is limited and heterogeneous, often constrained by short follow-up periods and varying outcome measures [45, 46]. In our study, the groups were based on whether or not they had participated in CPD in the past year, and the time allocated may be too short to obtain differences in some outcomes. One way to conceptualize nurses’ contributions to patient outcomes is through nursing-sensitive outcomes, which capture aspects of care particularly responsive to nursing practice. However, even within this framework, attributing changes in patient outcomes specifically to CPD is difficult, as nursing-sensitive outcomes are influenced by multiple interrelated factors, including organizational conditions, staffing levels, teamwork, and patient characteristics which complicates causal interpretations [47–50]. Studies examining relationships between nurses’ education or competence development and nursing-sensitive outcomes have therefore predominantly reported associative rather than causal relationships, with findings strongly shaped by organizational and contextual factors [48, 49]. When associations are observed, competence development is generally understood to exert its effects indirectly, through mediating mechanisms such as staffing adequacy, work environment, and team functioning, rather than through direct effects at the patient level [43]. Consequently, the effects of continuing professional development should not be expected to translate straightforwardly into differences in nurses’ satisfaction with given care. Instead, CPD may be more closely reflected in aspects of the work environment that support learning and professional interaction [51]. Consistent with this interpretation, nurses in the CPD group reported higher satisfaction with management and colleagues, including greater opportunities for discussion and reflection with peers. This may indicate a more learning-oriented organizational climate, where nurses continuously support, train, and provide feedback to one another in daily work, and where the workplace is experienced as a learning organization that actively fosters a learning climate. For the factors obstacles and preconditions, our results were non-significant. The absence of group differences in perceived obstacles and preconditions and job satisfaction may be explained by the fact that these aspects are largely determined by structural and organizational conditions, such as staffing adequacy, workload, work schedules, and leadership support, which are unlikely to be directly influenced by individual participation in CPD [52–54]. Previous research has consistently shown that job satisfaction and perceived work-related obstacles are shaped predominantly by system-level and organizational factors rather than by individual competence development [55, 56].

Our findings suggest that successful implementation of digital care requires not only technical competence, but also pedagogical support. The nurses reported a need for further skill development in delivering care through the digital tools, such as adapting communication strategies for chat and video consultations—an area that has become increasingly relevant with the introduction of new features in the Swedish SHD 1177 service. To maintain high-quality care in this rapidly evolving context, systematic education and training in digital care delivery are essential [57].

In our study, the participating nurses also identified structured feedback on their own calls and management of emotionally challenging interactions as important for professional development. These findings are consistent with previous research indicating that delivering telenursing care without adequate training can lead to discomfort and uncertainty for both nurses and patients, thereby compromising care quality and communication [57]. Rutledge and Gustin [57] further argue that nurses must be prepared for the expanding role of telehealth and emphasize that education for nurses should incorporate communication training, reflecting the preferences expressed by participants in our study. Advanced communication skills are required, especially when traditional visual or verbal cues are missing, as in telephone or chat consultations [58, 59]. Haakvort et al. [60] highlight the importance of individualized approaches, as factors influencing professional growth differ throughout a nurse’s career. The role of a nurse in telenursing settings is both knowledge-intensive and complex, constantly requiring the development of new professional, methodological, personal, and social competencies. The field of telenursing is constantly evolving and transforming. While most nurses were motivated to engage in CPD, it is of utmost importance that healthcare organizations recognize nurses’ individual goals and provide resources that support these varied needs.

### Methodological considerations and limitations

The design of the study was cross-sectional, which limits cause and effect associations.

The cross-sectional and context-specific design, together with the use of self-reported data, also limits causal interpretation and the generalizability of the results beyond the studied telenursing context. The findings should therefore be interpreted with caution. Another contextual limitation concerns differences in regulatory frameworks for CPD across countries. In settings where CPD is mandatory, participation may be driven by regulatory compliance rather than individual or organizational motivation, which may influence how CPD is experienced and its associations with work-related outcomes. This should be considered when interpreting the findings and in future comparative research. Validated instruments, see Table 1were used which is a strength. In one instrument used, the TWC one factor showed a Cronbach’s alpha slightly below the commonly recommended threshold of 0.70; however, McDonald’s omega for the same factor reached the acceptable level of 0.70. Given that omega is considered a more robust estimate of internal consistency, the reliability of this factor was deemed acceptable for the purposes of the present study [61]. The numbers of participants in the group of nurses not participating in CPD (*N* = 56) might be considered a bit small, which could reduce the statistical power of the analyses. The participants were predominantly women, which aligns with national statistics showing that women constitute approximately 88–90% o all registered nurses in Sweden, while men represent only about 10–12% o the nursing workforce [62].

## Conclusions

Participating in CPD during the previous year is associated with more positive work-related outcomes among telenursing nurses such as thriving, growth opportunities, better management and collegial support but not with nurse-sensitive outcomes such as nurse satisfaction with given care when compared to those who did not participate in CPD. Nurses described clear preferences and needs for clinically relevant, reflective, and self-directed professional development.

## Data Availability

The data presented, aggregated data, during the current study are available from the corresponding author upon reasonable request. Signing a data sharing agreement will be necessary. Individual data are unavailable due to General Data Protection Regulations (GDPR) and in accordance with the ethics application.

## Abbreviations

CPD: Continuing professional development
